# CT Findings in Acute, Subacute, and Chronic Ischemic Colitis: Suggestions for Diagnosis

**DOI:** 10.1155/2014/895248

**Published:** 2014-08-27

**Authors:** Francesca Iacobellis, Daniela Berritto, Dominik Fleischmann, Giuliano Gagliardi, Antonio Brillantino, Maria Antonietta Mazzei, Roberto Grassi

**Affiliations:** ^1^Department of Radiology, Second University of Naples, Piazza Miraglia 2, 80138 Napoli, Italy; ^2^Department of Radiology, Stanford University School of Medicine, 300 Pasteur Drive, Room S-072, Stanford, CA 94305-5105, USA; ^3^Emergency Department, “A. Cardarelli” Hospital, Via A. Cardarelli 9, 80131 Napoli, Italy; ^4^Department of Medical, Surgical and Neuro Sciences, Section of Radiological Sciences, University of Siena, Viale Bracci 10, 53100 Siena, Italy

## Abstract

*Purpose*. This paper aims at evaluating CT findings of occlusive and nonocclusive ischemic colitis (IC), in correlation with the etiology and the different phases of the disease. *Materials and Methods*. CT examination and clinical history of 32 patients with proven IC were retrospectively reviewed. The CT findings were analyzed according to the different phases of the disease (acute, subacute, and chronic). *Results*. Among the 32 CT examinations performed in the acute phase, 62.5% did not present signs of occlusion of the superior mesenteric artery (SMA) or inferior mesenteric artery (IMA), whereas IMA occlusion was detected in 37.5% of CT examinations. In the acute phase, the presence of pericolic fluid was found in 100% of patients undergoing progressive resorption from acute to subacute phase if an effective reperfusion occurred; the bowel wall thickening was observed in 28.1% patients in acute phase and in 86.4% patients evaluated in subacute phase. The unthickened colonic wall was found in all conditions where ischemia was not followed by effective reperfusion (71.9% of cases), and it was never found in chronic phase, when the colon appeared irregularly thickened. *Conclusion*. CT allows determining the morphofunctional alterations associated with the IC discriminating the occlusive forms from the nonocclusive forms. CT, furthermore, allows estimating the timing of ischemic damage.

## 1. Introduction

Ischemic colitis (IC) is the most common vascular disorder of gastrointestinal (GI) tract [1, 2] frequently seen in the elderly with a peak of incidence in the 7th decade [3].

IC is usually a form of nonocclusive (NO) ischemic disease without evidence of major artery or vein occlusion, even if sometimes it could have an arterial occlusive (O) etiology [4].

The ischemic injury may involve only the colonic mucosal and submucosal layer or result in transmural ischemic injury with high mortality requiring prompt surgery [5, 6].

Clinically, IC can be classified in two different forms, severe gangrenous (acute fulminant), accounting for 20.7% of cases, and nongangrenous (mild), representing about 79.3% of cases [7–10]. Nongangrenous IC can be divided into acute, subacute, and chronic types [1, 7, 8]. The incidence of nongangrenous forms is likely underestimated since clinical presentation is often nonspecific [9]. Common symptoms are hematochezia, persistent diarrhea, and abdominal pain [10–12].

Some authors consider colonoscopy as the test of choice for the diagnosis of IC; however, lower GI endoscopy is not without risk, especially in patients with severe colitis due to the risk of perforation [11, 13, 14]. For these reasons, imaging tests play an important role in the assessment and management of these patients, especially in acute phase (AP). CT scan with i.v. contrast media is the imaging technique of choice in acute patients being readily available in emergency department [1]; in suspected IC, the CT examination allows defining the injured colonic segment and detecting the presence of complications. Recently, investigations have suggested that magnetic resonance imaging (MRI) could play an important role especially in the follow-up of IC [15–17]. Considering previous experiences on animal models in which the correct evolution of the small and the large bowel ischemia/infarction was studied and defined [16, 18, 19], a similar evolution of IC can then be expected in humans in which the spectrum of findings remains currently not well understood [20].

Examining the previous consideration, the aim of our study was to evaluate the CT findings of IC in correlation with the etiology (O or NOIC etiology) and the different phases of the disease.

## 2. Patients and Methods

### 2.1. Study Design

This is a retrospective study conducted in two teaching hospitals.

Institutional Review Board approval and the informed consent from the patients were waived because of the retrospective nature of the study.

A computerized search of all medical records was used to identify 130 patients who were admitted with the suspected diagnosis of IC over a 5-year period (Jan 07–Jan 12). From these, 52 patients with acute arterial IC proven by endoscopy with biopsies or surgical pathology were considered for the enrollment in the present study. Among the 52 patients, only the patients that underwent at least one CT examination were enrolled. The patients enrolled were not affected by liver disease or other possible causes of ascites, so, 32 subjects (17 men and 15 women; median age 74, range 51–94 years) constituted the object of the analysis. Their medical history and CT examinations were retrospectively reviewed.

All the patients underwent CT examinations within 36 hours from the symptoms' onset (AP). Of the 32 patients, 10 did not receive further imaging studies after their first CT (4 underwent surgery for severe IC and 6 had conservative therapy for mild IC); the remaining 22 patients had a follow-up CT examination between 37 hours and 20 days (subacute phase: SP). Among these 22 patients, 12 patients did not receive further imaging studies after their second CT (3 underwent surgery due to the worsening of clinical picture and 9 were treated conservatively without further CT examinations). Ten patients underwent a third CT examination within 21 days and 2 months (chronic phase: CP) and were treated with conservative approach.

### 2.2. MDCT Technique

Abdominal CTs were obtained using a 4-detector row (4-r) scanner (Toshiba Aquilion) for 10 patients and using a 64-detector row (64-r) configuration (VCT, General Electric Healthcare, Milwaukee, Wis, USA) for the other 22 patients. In all patients, the examination was performed in supine position from the dome of the liver to the level of the perineum to cover the entire course of the intestine. All patients underwent unenhanced and contrast-enhanced CT, in the late arterial phase (start delay 45–50 seconds) and in the portal venous phase (start delay 70–80 seconds) following an i.v. injection of 2 mL/kg of nonionic contrast material (Iomeron 370; Bracco Diagnostics, Milan, Italy), followed by 40 mL of saline solution at a flow rate of 3-4 mL/s through an 18-gauge catheter placed into an antecubital vein using a power injector (SIAS 757, Bologna, Italy or MedRad Envision CT injector, MEDRAD Inc. Warrendale, PA, USA). Rectal air or rectal contrast material (cm) was not administered. The following technical parameters were used: in 4-r CT, 3.75 mm slice thickness at 2.5 mm reconstruction interval, tube voltage of 120–140 KVp, and reference mAs of 310 mA; in 64-r CT, effective slice thickness of 3.75 mm for plain acquisition, 1.25 mm in the late arterial phase, and 2.5 mm in the portal venous phase; beam pitch of 0.938, reconstruction interval of 0.8 mm, tube voltage of 120–140 KVp, and reference mAs of 250/700 mA were used. Automatic tube current modulation was used to minimize the radiation exposure. A standard reconstruction algorithm was used. Patients were instructed not to breathe during helical imaging to avoid motion artifacts.

### 2.3. Image Analysis and Comparison

CT examinations were evaluated in consensus by two radiologists (reader 1 and reader 2) experienced in gastrointestinal imaging (30 and 12 years of experience, resp.).

The following parameters were assessed: (a) findings of defects or occlusion of the superior mesenteric artery (SMA) or in the inferior mesenteric artery (IMA), (b) pericolic fluid, (c) peritoneal free fluid, (d) bowel wall hyperdensity at unenhanced CT, (e) hyperdensity with target configuration (two or three concentric rings) of colonic wall after i.v. contrast medium administration (cma), (f) presence/absence of bowel wall thickening (more than 3 mm in thickness), (g) shape of bowel thickness (uniform, nonuniform); (h) bowel wall hypodensity after i.v. contrast medium administration, (i) dilation of colonic lumen only gas-filled (diameter more than 5 cm), (j) parenchymal ischemia/infarction (liver/kidney/spleen), (k) wall pneumatosis, and (l) segmental or continuous colonic involvement, extent of the colon injury (A. right colon from cecum to left flexure, B. left colon from left flexure to sigma-rectum junction, C. left colon from left flexure to colon-sigma junction, and D. sigmoid colon).

During the review process, the radiologists were blinded to the phase of the disease and to pathological/surgical results. After the revision, the reported CT findings were analyzed according to the different phases (AP, SP, and CP).

## 3. Statistical Analysis

The differences between O IC and NO IC in each phase (AP, SP, and CP) were statistically analyzed and compared by means of Fisher's exact test. Values of *P* < 0.05 were considered statistically significant (Table 1).

## 4. Results

All the CT examinations presented a high diagnostic quality.

The analysis of the CT findings according to the different phases (AP, SP, and CP) is reported in Table 1.

In AP, among the 32 CT examinations, 20/32 patients (62.5%) had NO IC, whereas 12/32 (37.5%) had O IC.

In O IC, the following findings were detected: presence of pericolic fluid in 12/12 subjects (100%), peritoneal free fluid in 8/12 (66.7%), wall hyperdensity at the CT without i.v. cma in 5/12 (41.7%), uniform thickening of the injured colonic wall with target configuration after i.v. cma in 9/12 (75%), bowel wall hypodensity after i.v. cma in 3/12 (25%), colonic lumen dilation in 1/12 (8.3%), and no signs of parenchymal ischemia and/or wall pneumatosis in 12/12 (100%).

In NO IC, the following findings were detected: presence of pericolic fluid in 20/20 subjects (100%); peritoneal free fluid in 10/20 (50%); wall hyperdensity on the CT without i.v. cma in 9/20 (45%); colonic wall thickening with target configuration after i.v. cma in 0/20 (0%); bowel wall hypodensity after i.v. cma in 1/20 (5%); the colonic lumen dilated in 20/20 (100%); signs of parenchymal ischemia in 17/20 (85%), involving only the kidney in 5/20 (25%), only the liver in 4/20 (20%), only the spleen in 2/20 (10%), the liver and spleen in 2/20 (10%), the kidney and the spleen in 2/20 (10%), the kidney and the liver in 1/20 (5%), and the kidney, the liver, and the spleen in 1/20 (5%) patients; and wall pneumatosis in 1/20 (5%).

In SP, of the 22 CT examinations performed, 4 patients (18.2%) had O IC and 18 (81.8%) had NO IC.

In O IC, the following findings were detected: pericolic fluid in 4/4 patients (100%), peritoneal free fluid in 3/4 (75%), wall hyperdensity on the CT without i.v. cma in 1/4 (25%), uniform thickening of colonic wall with target configuration after i.v. cma in 1/4 (25%), bowel wall hypodensity after i.v. cma in 3/4 (75%), colonic lumen dilation in 3/4 (75%), and signs of parenchymal ischemia or pneumatosis in 0/4 (0%).

In NO IC, the following findings were detected: presence of pericolic fluid in 4/18 cases (22.2%); peritoneal free fluid in 0/18 (0%); wall hyperdensity on the CT without i.v. cma in 2/18 (11.1%); uniform thickening of the colonic wall with target configuration after i.v. cma in 18/18 (100%); bowel wall hypodensity after i.v. cma in 0/18 (0%); colonic lumen dilation in 0/18 (0%); signs of parenchymal ischemia in 8/18 (44.5%), involving only the kidney in 2/18 (11.1%), only the liver in 3/18 (16.7%), and only the spleen in 3/18 (16.7%) patients; and wall pneumatosis in 0/18 (0%).

In the CP, of 10 patients examined, one patient (10%) had O IC and 9 (90%) had NO IC. In O IC, the following findings were detected: pericolic and peritoneal free fluid in 0/1 (0%), wall hyperdensity on the CT without i.v. cma in 0/1 (0%), irregular thickening of the colonic wall without target configuration after i.v. cma in 1/1 (100%), bowel wall hypodensity after i.v. cma in 0/1 (0%), the colonic lumen dilation in 1/1 (100%), and signs of parenchymal infarction or pneumatosis in 0/1 (0%).

In NO IC, the following findings were detected: pericolic and peritoneal free fluid in 0/9 (0%); wall hyperdensity on the CT without i.v. cma in 0/9 (0%); irregular thickening without target configuration of the colonic wall after i.v. cma in 9/9 (100%); bowel wall hypodensity after i.v. cma in 0/9 (0%); colonic lumen dilation in 6/9 (66.7%) and normal colonic caliber in 3/9 (33.3%); and signs of parenchymal infarction in 4/9 (44.4%), involving the liver in 3/9 (33.3%) and the spleen in 1/9 (11.1%); wall pneumatosis in 0/9 patients (0%).

In NO IC, the colonic involvement included the following: A in 20/20 (100%) of cases, A + B in 10/20 (50%) of cases, and A + C in 4/20 (20%) of cases.

In O IC, the colonic involvement included the following: B in 2/12 (16,7%), C in 5/12 (41,65%), and D in 5/12 (41,65%).

Statistically significant differences *P* < 0.001 (Table 1) between O IC and NO IC were found for the following findings of AP: hyperdensity with target configuration of colonic wall after i.v. cma, presence of bowel wall thickening, shape of bowel thickness, dilation of colonic lumen only gas-filled, and parenchymal ischemia/infarction.

## 5. Discussion

Diagnosis of IC is based on a combination of clinical suspicion, radiological, endoscopical, and histological findings.

The CT scan, performed before and after i.v. contrast media, allows defining the diagnosis and the location of injury excluding other serious medical conditions [1, 21, 22].

The most common cause of IC is low flow state [23] and so some authors consider IC as a form of nonocclusive ischemic disease (NOMI). Many medical conditions as myocardial infarction, congestive heart failure, aortic insufficiency, and renal or hepatic disease, and also some medications, can cause ischemia by reducing blood flow to the colon [4, 8]. In our series, NOMI accounted for 20/32 patients (62.5%) [23]. The diagnosis of NOMI was based on the lack of IMA and SMA occlusion associated with clinical history of low flow states [9, 24–26].

In a minor percentage of cases, IC can be due to occlusive causes (37.5% in our series) as IMA thrombosis or embolism [27]. The IMA occlusion is detected at enhanced CT as luminal defect [28]. Hypertension, diabetes mellitus, ischemic heart disease, congestive heart disease, age, and hyperlipidemia are known risk factors [29], as well as renal failure [9–11].

From the data analysis, we can summarize the CT findings of IC as occurring in AP, SP, and CP.

In AP, the intestinal ischemic damage was always related to the presence of pericolic fluid, which, in 100% of patients, was located in paracolic recess, due to the peritoneal response to the colonic ischemic injury. This finding are useful in diagnosing the IC before reperfusion, being the only sign beyond dilation only gas-filled and “paper-thin wall”. However, the latter could be misdiagnosed due to the similarity with the physiological distension of the colon caused by the presence of intestinal gas.

The pericolic fluid may undergo progressive resorption from AP to SP if an effective reperfusion occurs. These findings were also observed and confirmed in an animal model of IC [16]. Peritoneal free fluid was present in 56.3% of patients in AP.

**Figure 1:**
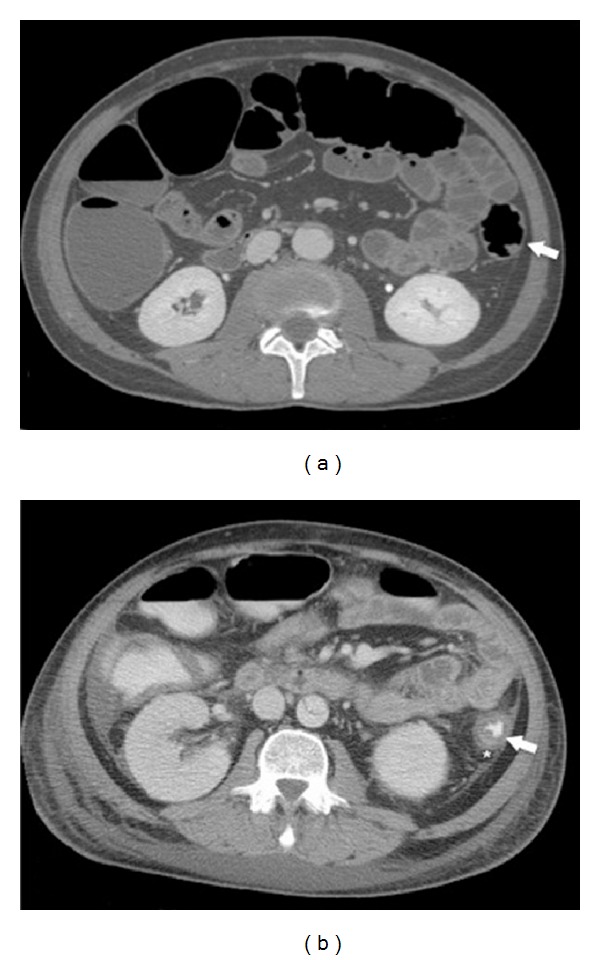
65-year-old patient with abdominal aortic dissection in acute phase IC caused by NOMI: (a) before reperfusion, colonic wall appears thinned (paper-thin wall) and hypotonic (arrow); (b) after reperfusion, note colonic wall thickening (arrow) and pericolic fluid (star); at endoscopic evaluation (not showed), the colon shows parcelled necrosis confirming the diagnosis of NOMI with reperfusion.

The unthickened or “paper-thin” colonic wall was found in all conditions where ischemia was not followed by effective reperfusion [30]; in AP, this condition was observed in all cases of NOMI (62.5%) before reperfusion (Figure 1) and in vasculopathic patients with IMA occlusion in which Riolan's arcade is unable to provide for an adequate blood supply (9.4%). A low flow state causes a broad reduction of blood flow, simulating a simultaneous obstruction of SMA and IMA; this explains the finding of wall thickness from normal to “paper-thin wall” with gaping lumen that we observed in AP of NO IC [25, 31]. Thinning of the bowel wall or “paper-thin wall” is caused by volume loss of tissue and vessels in the bowel wall and by the loss of intestinal muscular tone due to the lack of blood flow [32, 33].

The i.v. contrast media allows the evaluation of the bowel wall enhancement. In AP, there was bowel wall hypodensity on enhanced CT in 12.5% of patients. The bowel wall hypodensity in this case was related to the absence of effective reperfusion due to the bowel wall ischemic injury.

In case of NOMI, beyond intestinal damage, there was also a reduction of contrast enhancement of abdominal parenchyma, most commonly in the spleen, kidneys, and liver, which was observed in 85% of our case history. In AP, the most affected organ was the kidney, injured in 9/17 (52.9%) patients, followed by the liver, in 8/17 (47%) patients, and the spleen, in 7/17 (41.2%) patients.

**Figure 2:**
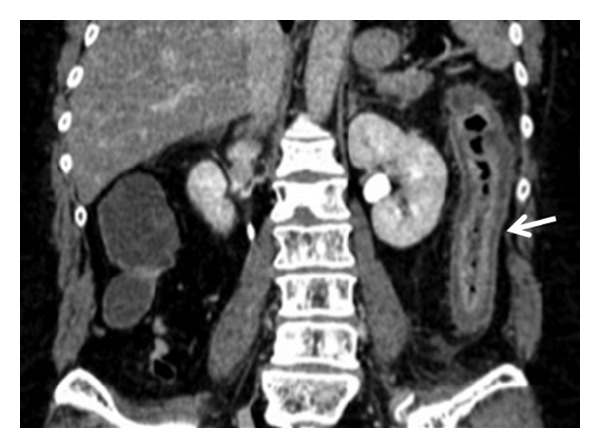
Contrast-enhanced CT in patients with acute occlusive IC: homogeneous left colonic involvement with disappearance of the lumen (arrow) and hyperperfusion of the mucosa in coronal plane. The endoscopy showed the presence of thickened mucosa with hemorrhagic phenomena. The histological findings were represented by increased thickness of the entire mucosal layer with intraparietal edema with hemorrhage and reactive inflammatory cells.

**Figure 3:**
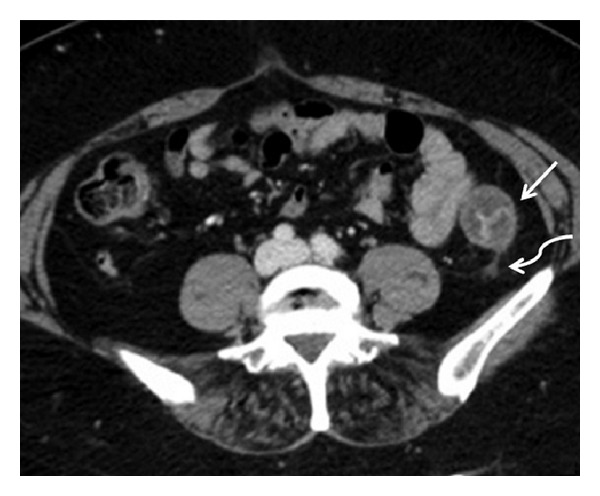
Patients with embolic IMA occlusion in acute phase: left colonic wall thickening (white arrow) with evidence of “little rose” sign or target aspect after i.v. contrast medium administration was detected; pericolic fluid was also present (curved arrow); the endoscopy showed edematous and thickened mucosa with diffuse petechial hemorrhage and hemorrhagic nodules; in some segments also hemorrhagic ulcerations were detected. These findings were histologically related to vascular congestion with intraparietal haemorrhage and increased cellularity with the presence of reactive inflammatory cells.

As reported in literature, most frequently in acute IC, colonic wall appears thickened because of mural edema or hemorrhage [1, 5, 8, 34, 35]; in our series, we found this finding only in 28.1% patients observed in AP. This is related to the etiopathogenesis of the disease, which, in the AP, was due to a colonic acute ischemic injury caused by IMA occlusion rapidly followed by reperfusion of the involved tract from Riolan's arcade's collateral vessels (Figure 2). The unenhanced phase may detect any hemorrhagic phenomenon of the bowel wall; in our patients, this finding was observed as bowel wall hyperdensity in 43.8% of patients in AP. Mucosal hyperdensity from hemorrhagic phenomena may determine a typical feature, the “little rose” sign [34] (Figure 3). In AP, pneumatosis was detected as late and severe sign of bowel infarction in only one patient.

In SP, the pericolic and the free peritoneal fluid may increase or decrease depending on the evolution of the ischemic damage. If there is no reperfusion, the ischemic damage progresses, the bowel wall remains hypodense, unthickened, or thinned, and the fluid increases: this condition was observed in 15% of our patients.

**Figure 4:**
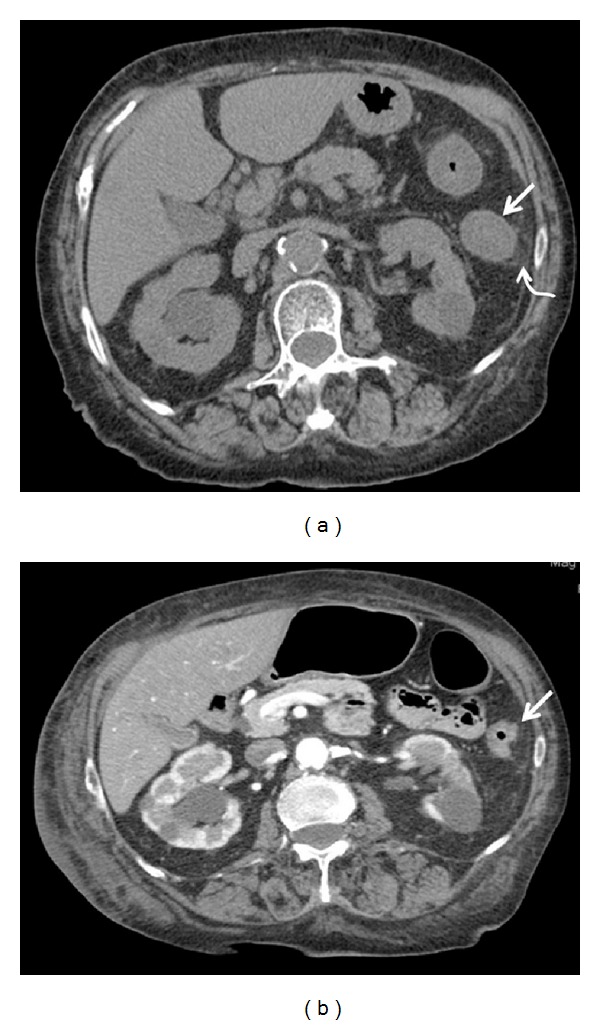
Appearance of ischemic colon after reperfusion: when colonic reperfusion occurs, the bowel wall becomes thickened (a, straight arrow) and pericolic fluid is detected (a, curved arrow); if the reperfusion is effective (b) these findings progressively decrease and the damage is restored.

If the reperfusion occurs, the bowel wall becomes thickened with target sign and the pericolic and free peritoneal fluid may progressively decrease if the damage is restored, as observed in 86.4% (19/22) of our cases (Figure 4).

**Figure 5:**
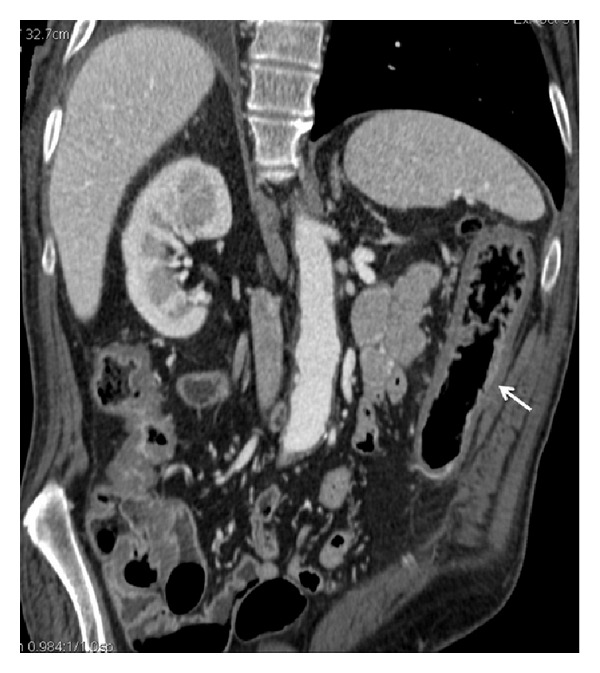
Continuous mild and irregular circumferential thickening of the colon with gaping lumen (arrow) in the chronic phase of IC. At endoscopy, the colon appeared with gaping lumen and disappearance of the haustral folds; the mucosa was pale and irregularly thickened. The histological examination showed the presence of areas of fibrotic tissue.

In CP, pericolic fluid was never found; in the injured colonic wall, fibrotic reaction develops leading to continuous mild and irregular circumferential thickening with gaping lumen [18, 19], as observed in 100% of patients, and so, the target sign, due to acute mucosal hemorrhagic and edematous phenomena, is absent (Figure 5).

In our series, as already reported in literature [16, 24], ischemic changes were related to vascular anatomy, always uniformly distributed in the injured colonic segment and markedly distinct to the nonischemic tract. These remarks are useful in considering differential diagnosis with pathology having similar imaging features but different distributions, as Crohn or other inflammatory conditions [12].

Regarding the colonic involvement in ischemic damage, we observed that, in the case of NOMI, the right colon was always injured with or without left colon participation, 70% (14/20) and 30% (6/20), respectively. In case of IMA occlusion, the right colon was never involved and the left colon was injured from left flexure to sigma-rectum junction in 16.7% (2/12), the left colon from left flexure to descending colon-sigma in 41.7% (5/12), and the sigmoid colon in 41.7% (5/12).

This work has some limitations: the first one is the retrospective nature of the study, which may determine a bias in the data analysis and the second is the modest number of the patients analyzed.

In conclusion, the results of this study showed that CT allows determining the morphofunctional alterations associated with the IC and allows estimating the timing of ischemic damage.

- In AP, the main differential finding between O IC and NO IC is represented by the bowel wall thickening and related signs as uniform shape and target sign.
- In SP, it is important to evaluate the effectiveness of reperfusion; in this sense, the changes in the bowel wall thickness and in the free and pericolic fluid amount are useful parameters to be monitored.
- In CP, it is no longer important to define the etiology but the effects of the ischemic injury, represented by the nonuniform fibrosis of the bowel wall.

Particular attention should be paid to the nonocclusive forms (NOMI) before reperfusion representing the more difficult form of IC to detect at imaging since the only paper-thin wall can be misdiagnosed like normal colon; in this case, the presence of pericolic fluid may suggest the presence of pathology together with the suspicion induced by medical history [6]. Diagnostic difficulties may also be encountered in subacute forms where the colon wall thickening could be misdiagnosed as normal wall with collapsed lumen and in chronic forms where the irregular thickening of large bowel could be misdiagnosed if the patient's clinical history is unknown.

## Key Points

- CT allows estimating the timing of damage in patients with ischemic colitis.
- CT allows distinguishing between reperfused and nonreperfused colon.
- Nonocclusive forms without or before reperfusion are the most difficult forms to diagnose.

## Figures and Tables

**Table 1:** CT findings of IC according to the different phases of disease and etiology.

	Acute phase (32)			Subacute phase (22)			Chronic phase (10)		
	Occlusive (12)	Nonocclusive (20)	*P*	Occlusive (4)	Nonocclusive (18)	*P*	Occlusive (1)	Nonocclusive (9)	*P*
a	12/12 (100%)	20/20 (100%)	—	4/4 (100%)	4/18 (22.2%)	0.01	0/1	0/9	—
b	8/12 (66.7%)	10/20 (50%)	—	3/4 (75%)	0/18	0.003	0/1	0/9	—
c	5/12 (41.7%)	9/20 (45%)	—	1/4 (25%)	2/18 (11.1%)	—	0/1	0/9	—
d	9/12 (75%)	0/20	**<0.001**	1/4 (25%)	18/18 (100%)	0.003	0/1	0/9	—
e	9/12 (75%)	0/20	**<0.001**	1/4 (25%)	18/18 (100%)	3	1/1 (100%)	9/9 (100%)	—
f	9/12 (75%)	0/20	**<0.001**	1/4 (25%)	18/18 (100%)	0.003	0/1	0/9	—
g	3/12 (25%)	1/20 (5%)	—	3/4 (75%)	0/18	3	0/1	0/9	—
h	1/12 (8.3%)	20/20 (100%)	**<0.001**	3/4 (75%)	0/18	0.003	1/1 (100%)	6/9 (66.7%)	—
i	0/12	17/20 (85%)	**<0.001**	0/4	8/18 (44.5%)	—	0/1	4/9 (44.4%)	—
j	0/12	1/20 (5%)	—	0/4	0/18	—	0/1	0/9	—

The differences between occlusive and nonocclusive forms in each phase were analyzed and compared by means of Fisher's exact test. Values of *P* < 0.05 (bold) were considered statistically significant.

(pt) patients; (a) pericolic fluid; (b) peritoneal free fluid; (c) bowel wall hyperdensity at unenhanced CT; (d) hyperdensity with target configuration (two or three concentric rings) of colonic wall after i.v. contrast medium administration; (e) presence of bowel wall thickening (more than 3 mm in thickness); (f) shape of bowel thickness (uniform); (g) bowel wall hypodensity after i.v. contrast medium administration; (h) dilation of colonic lumen only gas-filled (diameter more than 5 cm); (i) parenchymal ischemia/infarction (liver and/kidney/spleen); (j) wall pneumatosis.

## References

[1] Theodoropoulou A, Koutroubakis IE (2008). Ischemic colitis: clinical practice in diagnosis and treatment. *World Journal of Gastroenterology*.

[2] Higgins PDR, Davis KJ, Laine L (2004). Systematic review: The epidemiology of ischaemic colitis. *Alimentary Pharmacology and Therapeutics*.

[3] Sotiriadis J, Brandt LJ, Behin DS, Southern WN (2007). Ischemic colitis has a worse prognosis when isolated to the right side of the colon. *American Journal of Gastroenterology*.

[4] Balthazar EJ, Yen BC, Gordon RB (1999). Ischemic colitis: CT evaluation of 54 cases. *Radiology*.

[5] Alturkistany S, Artho G, Maheshwari S, Blaichman J, Kao E, Mesurolle B (2012). Transmural colonic ischemia: clinical features and computed tomography findings. *Clinical Imaging*.

[6] Brandt LJ, Feuerstadt P, Blaszka MC (2010). Anatomic patterns, patient characteristics, and clinical outcomes in ischemic colitis: a study of 313 cases supported by histology. *The American Journal of Gastroenterology*.

[7] Scowcroft CW, Sanowski RA, Kozarek RA (1981). Colonoscopy in ischemic colitis. *Gastrointestinal Endoscopy*.

[8] Brandt LJ, Boley SJ (1992). Colonic ischemia. *Surgical Clinics of North America*.

[9] Gore RM, Yaghmai V, Thakrar KH (2008). Imaging in intestinal ischemic disorders. *Radiologic Clinics of North America*.

[10] Chang HJ, Chung CW, Ko KH, Kim JW (2011). Clinical characteristics of ischemic colitis according to location. *Journal of the Korean Society of Coloproctology*.

[11] Elder K, Lashner BA, Al Solaiman F (2009). Clinical approach to colonic ischemia. *Cleveland Clinic Journal of Medicine*.

[12] Glauser PM, Wermuth P, Cathomas G, Kuhnt E, Käser SA, Maurer CA (2011). Ischemic colitis: clinical presentation, localization in relation to risk factors, and long-term results. *World Journal of Surgery*.

[13] Danse EM, van Beers BE, Jamart J (2000). Prognosis of ischemic colitis: Comparison of color doppler sonography with early clinical and laboratory findings. *The American Journal of Roentgenology*.

[14] Beppu K, Osada T, Nagahara A (2011). Relationship between endoscopic findings and clinical severity in ischemic colitis. *Internal Medicine*.

[15] Kim MY, Suh CH, Kim ST (2004). Magnetic resonance imaging of bowel ischemia induced by ligation of superior mesenteric artery and vein in a cat model. *Journal of Computer Assisted Tomography*.

[16] Iacobellis F, Berritto D, Somma F (2012). Magnetic resonance imaging: a new tool for diagnosis of acute ischemic colitis?. *World Journal of Gastroenterology*.

[17] Mazzei MA, Guerrini S, Squitieri NC (2013). Magnetic resonance imaging: Is there a role in clinical management for acute ischemic colitis?. *World Journal of Gastroenterology*.

[18] Berritto D, Somma F, Landi N (2011). Seven-Tesla micro-MRI in early detection of acute arterial ischaemia: Evolution of findings in an in vivo rat model. *Radiologia Medica*.

[19] Somma F, Berritto D, Iacobellis F (2013). 7T *μ*MRI of mesenteric venous ischemia in a rat model: timing of the appearance of findings. *Magnetic Resonance Imaging*.

[20] Romano S, Lassandro F, Scaglione M, Romano L, Rotondo A, Grassi R (2006). Ischemia and infarction of the small bowel and colon: Spectrum of imaging findings. *Abdominal Imaging*.

[21] Mazzei MA, Guerrini S, Squitieri NC, Genovese EA, Mazzei FG, Volterrani L (2012). Diagnosis of acute mesenteric ischemia/infarction in the era of multislice CT. *Recenti Progressi in Medicina*.

[22] Taourel P, Aufort S, Merigeaud S, Doyon FC, Hoquet MD, Delabrousse E (2008). Imaging of ischemic colitis. *Radiologic Clinics of North America*.

[23] Baixauli J, Kiran RP, Delaney CP (2003). Investigation and management of ischemic colitis. *Cleveland Clinic Journal of Medicine*.

[24] Thoeni RF, Cello JP (2006). CT imaging of colitis. *Radiology*.

[25] Romano S, Romano L, Grassi R (2007). Multidetector row computed tomography findings from ischemia to infarction of the large bowel. *European Journal of Radiology*.

[26] Mazzei MA, Guerrini S, Cioffi Squitieri N, Imbriaco G, Mazzei FG, Volterrani L (2014). Non-obstructive mesenteric ischemia after cardiovascular surgery: not so uncommon. *Annals of Thoracic and Cardiovascular Surgery*.

[27] Park CJ, Jang MK, Shin WG (2007). Can we predict the development of ischemic colitis among patients with lower abdominal pain?. *Diseases of the Colon and Rectum*.

[28] Cappell MS, Mahajan D, Kurupath V (2006). Characterization of ischemic colitis associated with myocardial infarction: an analysis of 23 patients. *The American Journal of Medicine*.

[29] Sun MY, Maykel JA (2007). Ischemic colitis. *Clinics in Colon and Rectal Surgery*.

[30] Saba L, Berritto D, Iacobellis F (2013). Acute arterial mesenteric ischemia and reperfusion: macroscopic and MRI findings, preliminary report. *World Journal of Gastroenterology*.

[31] Mazzei MA, Mazzei FG, Marrelli D (2012). Computed tomographic evaluation of mesentery: diagnostic value in acute mesenteric ischemia. *Journal of Computer Assisted Tomography*.

[32] Ripollés T, Simó L, Martínez-Pérez MJ, Pastor MR, Igual A, López A (2005). Sonographic findings in ischemic colitis in 58 patients. *American Journal of Roentgenology*.

[33] Furukawa A, Kanasaki S, Kono N (2009). CT diagnosis of acute mesenteric ischemia from various causes. *American Journal of Roentgenology*.

[34] Macari M, Balthazar EJ (2001). CT of bowel wall thickening: significance and pitfalls of interpretation. *American Journal of Roentgenology*.

[35] Wiesner W, Khurana B, Ji H, Ros PR (2003). CT of acute bowel ischemia. *Radiology*.

